# Pesticide-related illness reported to and diagnosed in Primary Care: implications for surveillance of environmental causes of ill-health

**DOI:** 10.1186/1471-2458-9-219

**Published:** 2009-07-06

**Authors:** Lesley Rushton, Vera Mann

**Affiliations:** 1 Department of Epidemiology and Public Health, Imperial College London, Faculty of Medicine, Norfolk Place, London, W2 1PG, UK; 2 Medical Statistics Unit, London School of Hygiene and Tropical Medicine, London, WC1E 7HT, UK

## Abstract

**Background:**

In Great Britain (GB), data collected on pesticide associated illness focuses on acute episodes such as poisonings caused by misuse or abuse. This study aimed to investigate the extent and nature of pesticide-related illness presented and diagnosed in Primary Care and the feasibility of establishing a routine monitoring system.

**Methods:**

A checklist, completed by General Practitioners (GP) for all patients aged 18+ who attended surgery sessions, identified patients to be interviewed in detail on exposures and events that occurred in the week before their symptoms appeared.

**Results:**

The study covered 59320 patients in 43 practices across GB and 1335 detailed interviews. The annual incidence of illness reported to GPs because of concern about pesticide exposure was estimated to be 0.04%, potentially 88400 consultations annually, approximately 1700 per week. The annual incidence of consultations where symptoms were diagnosed by GPs as likely to be related to pesticide exposure was 0.003%, an annual estimate of 6630 consultations i.e. about 128 per week. 41% of interviewees reported using at least one pesticide at home in the week before symptoms occurred. The risk of having symptoms possibly related to pesticide exposure compared to unlikely was associated with home use of pesticides after adjusting for age, gender and occupational pesticide exposure (OR = 1.88, 95% CI 1.51 – 2.35).

**Conclusion:**

GP practices were diverse and well distributed throughout GB with similar symptom consulting patterns as in the Primary Care within the UK. Methods used in this study would not be feasible for a routine surveillance system for pesticide related illness. Incorporation of environmental health into Primary Care education and practice is needed.

## Background

Pesticides, pesticide products and related chemicals have been found to have a wide range of health effects. They include: mutagenic substances, carcinogens or probable carcinogens, endocrine disrupters, reproductive toxic substances and neurotoxic substances [1,2]. The effect of low-level, long-term exposure has been of recent concern, with the organophosphate pesticides as a group receiving a great deal of medical research interest, particularly with regard to their potential effects on farmers using sheep dips [2-4]. Although pesticides have undoubted acute health effects, these usually occur as a result of accidental or deliberate misuse. In the UK, information on acute events can be obtained, for example, from the NHS Hospital Episode Statistics [5]. Much less is known about the incidence of ill-health due to low-levels of pesticide exposure and there are currently no surveillance schemes in Primary Care in Great Britain that identify an illness as possibly due to pesticide exposure, whether occupational or environmental.

The aim of this study was to fill this gap in knowledge and in particular to: estimate the annual national incidence and prevalence of pesticide-related illness presented to and diagnosed by General Practitioners (GP); investigate associations between symptoms and possible pesticide exposure, evaluate the feasibility and practicalities of setting up permanent arrangements to collect data on pesticide related illness in Primary Care. As few patients (92: 7% of interviewees) reported occupational use of pesticides, this paper focuses on results related to home and environmental pesticide exposure, a fuller technical report is available elsewhere [6].

There are several challenges in surveillance of pesticide related illness in general practice including the large number of symptoms (often rather vague) that could potentially result from low-level exposure to pesticides and the lack of readily available sensitive, specific and interpretable biological tests to confirm exposure. Most GPs, presented with a patient reporting such symptoms in the absence of reported exposure to pesticides, do not routinely consider the possibility of pesticide exposure. Any surveillance system must therefore encourage the GP to consider further a possible relationship but without over-prompting. The project thus focussed on patients who consulted to report recent pesticide exposure, with or without current symptoms, and on patients who presented with symptoms that were 'unusual' for them i.e. a new occurrence and not a chronic recurring problem and that the GP considered could potentially be related to a (possible) recent pesticide exposure. Although it was thought that acute severe illness related to high exposure was likely to lead to presentation and treatment in secondary care rather than primary care it was also thought important to capture any acute symptoms.

## Methods

The study was carried out between 2003 and 2006 in general practices from the UK General Practice Research Framework, an organisation of almost 1100 general practices throughout the UK involved in epidemiological and health service research [7]. The GPRF network covers over 9% of UK practices, giving access to 12% of the population, with sufficient number of all types and in all areas to provide representative samples of the UK practices with regard to demographic characteristics and agricultural practices. Of particular relevance to this project is that 14% of the practices are in areas classified by the Office of National Statistics as remote rural and 17% are in mixed urban/rural areas. Information about the study and an invitation to participate was sent to all GPs on the GPRF database. Each participating GP completed a one page screening checklist for all patients aged 18 years or over consulting during a surgery session. Our pilot study indicated that GPs would be unwilling to use the screening checklist at all surgery sessions. They were therefore requested to do this for at least 2 sessions per week during a year of data collection. The practice research nurse organised a continuous flow of both checklists and interviews throughout the data collection period assuring that the consultation sessions occurred on different days and in both mornings and afternoons to ensure representation of patient consulting patterns (e.g. not always on Monday mornings or Friday afternoons when more acute or urgent consultations might take place).

An information pack was provided for GPs on pesticide-related illnesses with instructions on how to complete the checklist. The research nurses attended training days and, during the project, back-up training or additional support was provided by GPRF regional training nurses who were also responsible for quality control during the study ensuring standardisation and checking that the practice was fulfilling all the requirements of research governance. GPs were asked to carry out their 'normal' consulting practice and to complete the checklists at the end of the consultation.

The checklist was used to identify patients who attended, with or without reporting symptoms, because of their concern about exposure to pesticides and those patients consulting with symptoms that were unusual for them. On each checklist GPs were asked to record their opinion of whether the patient's symptoms were likely, possibly, unlikely to be or definitely not related to pesticide exposure. A computer algorithm selected patients who were eligible for an invitation to an in-depth interview with the research nurse if:

• They consulted because of their concern about exposure to pesticides regardless of the opinion of the GP about potential relation to pesticide exposure.

• They had serious acute symptoms such as blurring of vision, vertigo, respiratory compromise that were not definitively attributed by the GP to a cause other than pesticides exposure

• They had newly occurring flu type, respiratory, gastro-intestinal, skin, eye or neurological symptoms, which were unusual for the patient and were not definitively attributed by the GP to a cause other than pesticides exposure

• They had the above symptoms which were not unusual, i.e. recurring symptoms, for the patient but which the GP thought were likely or possibly related to pesticide exposure

Initially ethical approval was only given to approach eligible patients about an interview by a single letter without a reminder. As about 40% of the eligible patients in the pilot study did not respond to the invitation to attend for interview, the Chairman of the Ethics Committee was approached and approval was obtained for sending reminder invitation letters to non-responders. Later permission was also obtained to carry out the interview over the telephone although this method was seldom preferred by patients.

The computer-based interview questionnaire consisted of sections on:

1. Occupational exposure, including applying and mixing pesticides during work, formulations, frequency and duration of potential exposure, substance(s) including chemical and/or brand name and use of personal protective equipment.

2. Amateur use at home and in the garden including the use and mixing of pesticides, occurrences of professional pest control in the home and the storage and disposal of pesticides at home.

3. Hobbies and leisure during which exposure to hazardous materials or pesticides might have occurred.

4. Other suspected exposure to pesticides, including incidents such as accidental exposures from spray drift and incidents occurring in public places and near farmland.

5. Demographic, medical and miscellaneous information

All the questions focussed on exposures and events that occurred in the week before the symptoms appeared. Patients were asked about the numbers of days' use of pesticides and whether this was more use than usual. Information on use of other substances in the home that potentially might lead to similar symptoms as those expected from pesticides (e.g. detergents, solvents, paints, etc.) and whether they had been used more frequently, in a larger quantity or with a change of brand was also collected.

Before the main study, a pre-pilot phase was carried out in Northern Ireland to test the feasibility of the GP administered checklist followed by a pilot study in 9 practices in England and Wales to pilot both the use of the checklist and the interview questionnaire (see additional file 1). Some changes were made for the main study to improve accuracy of responses, to ensure questions could not be omitted and to improve clarity.

To ensure that duplicate interviews did not take place nurses were instructed not to invite a patient more than once if the patient had consulted more than once within a short period of time for the same illness and more than one checklist had been completed. However, a consultation could be considered as a separate episode if there was a gap of at least two weeks between consultations and it was clearly for a new problem.

Ethical approval was obtained through the UK Multi-centre Research Ethics Committees (Wales) and research governance approval was obtained from all the relevant Primary Care Organisations.

Statistical analyses were carried out using statistical software Stata version 9.2. Estimations of annual incidence proportion (cumulative incidence) and prevalence (proportion) of pesticide-related illness presented to GPs used the total number of patients screened as denominator, while the estimations of incidence and prevalence of possibly or likely pesticide-related illness diagnosed by GPs, without the patients specifically mentioning exposure, excluded the number of patients who reported exposure from the denominator. Annual incidence estimations included only patients with newly occurring symptoms in the nominators; annual prevalence estimations also included those who presented with recurring symptoms. Ninety five percent confidence intervals for incidence and prevalence were estimated using the normal approximation or the exact method if the numbers were small.

Statistical methods included descriptive analyses together with univariable and multivariable logistic regressions to assess the association between risk factors and potential pesticide-related illness using robust standard error estimation to take account of clustering of patients within GP practices.

## Results

### Participating practices

157 GPs and 7 nurse practitioners participated from 43 practices between November 2004 and July 2006 (not necessarily all GPs in a participating practice). Six practices withdrew within 4 months or less. Reasons given for withdrawal included illness of the research nurse or GP, changes of GPs resulting in replacements being unwilling to participate and heavy practice workloads. The majority carried out the study for well over 6 months giving an average of 52.6 weeks. The number of checklists completed per session was generally quite consistent between practices, varying between 6 and 14 giving an average of 11 per session.

**Figure 1:**
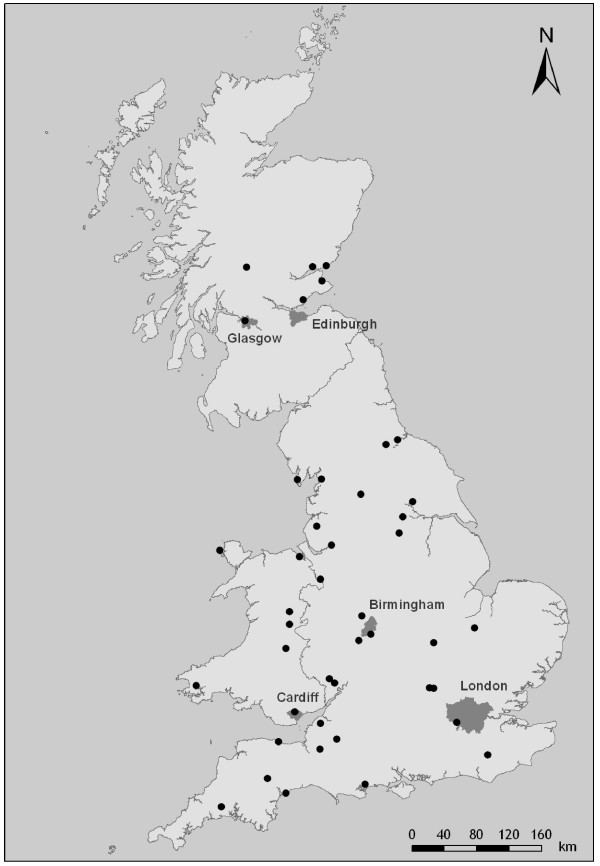
**Locations of the participating General Practices Locations of the participating General Practices**.

The practices were spread throughout GB; industrial areas (5), cities or urban areas including outer London and metropolitan districts (13), mixed urban and rural areas including new towns and coastal resorts (12), rural areas (13) (Figure 1). The number of partners in the practices ranged from 1 to 8 or more, with practice sizes ranging from about 4000 patients to over 13000.

### Eligibility for and response to interview invitation

59320 checklists (male patients 40.4%) were completed during 5446 GP surgery sessions. Nearly 25% (14490) of patients were asymptomatic and consulting the GP for other reasons and one patient, though asymptomatic, consulted the GP because of exposure to pesticides. 4741 (8%) patients were identified as eligible for invitation for interview, all of whom were received an invitation. 1335 patients (28.2% of those eligible and 49.8% of those who did not refuse) completed the interview, 2060 (43.5%) refused to be interviewed and 1346 (28.4%) had not replied by the end of the study. The distributions of symptoms were similar in all three groups regardless whether the patients agreed, refused to be interviewed or did not respond. On average, non-responders and those who refused to be interviewed were slightly younger than those who were not eligible and those who completed the interview (mean ages 46.3, 51.1, 54.2, 56.7 years respectively). Similar proportions of men and women were eligible for an interview (8.7%, 7.5% respectively). Of those who were eligible proportions who refused to be interviewed were similar for men (44%) and women (43%) as were proportions who did not respond (29.1% men, 27.8% women).

### Incidence and prevalence of symptoms related to pesticide exposure

Forty two people consulted because of their concern about exposure to pesticides. The GP thought the symptoms were likely to be related to exposure to pesticides for 13 of these patients and also for a further 7 patients who did not report exposure (Table 1).

**Table 1:** General Practitioner's opinion on likelihood of symptoms being related to pesticides

**Reason for Consultation**	**New or Recurring Symptoms**	**GP's opinion on likelihood of symptoms being pesticide-related**				
		**Likely**	**Possible**	**Unlikely**	**Definitely not**	**Total**

**Because of concern about pesticide exposure**	**Asymptomatic**	0	0	0	0	1
	**Recurring**	2	4	6	1	13
	**Newly occurring**	10	11	3	0	24
	**Not known**	1	3	0	0	4
	**Total**	13	18	9	1	42


**Pesticide exposure not mentioned by patient**	**Asymptomatic**	0	0	0	0	14490
	**Recurring**	5	528	13451	20207	34191
	**Newly occurring**	2	972	4165	4020	9159
	**Not known**	0	81	932	425	1438
	**Total**	7	1581	18548	24652	59278


**Overall total**		20	1599	18557	24653	59320

GPs also thought that 1599 patients (2.7% overall) had symptoms that were possibly related to pesticide exposure (33.7% of all eligible patients). 18557 patients (31.3% overall) were thought by the GP to have symptoms that were unlikely to be related to pesticide exposure, 3120 of these patients were eligible for in-depth interview (65.8% of all eligible patients).

As the average number of weeks for which the study was carried out was approximately a year, no weighting was carried out for the prevalence and incidence estimation. The denominator for estimating the annual incidence and prevalence for pesticide-related illness presented to GPs was thus the total number of patients screened (59320) and for pesticide-related illness diagnosed by GPs it was 59278 i.e. excluding the 42 patients who reported exposure to pesticides (Table 1).

The annual prevalence of consultations because of concern by the patient about pesticide exposure is estimated to be 0.07% (42/59320) (95% CI 0.05%, 0.09%) and the estimate of the annual incidence (i.e. new cases) is 0.04% (24/59320) (95% CI 0.02%, 0.06%) (Table 1). For people who did not consult because of concern about exposure to pesticides, the estimate of the annual prevalence of consultations for which the GP thought the symptoms were likely to be related to pesticide exposure is extremely small, 0.01% (7/59278) (95% exact CI 0.004%, 0.02%), with an estimate of those with symptoms thought by the GP to be possibly related to pesticide exposure being 2.7% (1581/59278) (95% CI 2.5%, 2.8%). The annual incidence of consultations for which the GP though symptoms were likely to be related to pesticide exposure is 0.003% (2/59278) (95% exact CI 0%, 0.01%) and the annual estimate of those with symptoms thought by GP to be possibly related to pesticide exposure is 1.6% (972/59278) (95% CI 1.5%, 1.7%) respectively.

In 2001, GPs in the UK carried out about 221 million consultations (patients aged 16 years or more), [8]. Although our study was based on people aged 18 years or more in GB (excluding Northern Ireland) the estimate of an annual incidence of 0.04% for consultations made by patients because of concern about pesticides translates to an annual estimate of 88400 consultations i.e. approximately 1700 per week for people aged 16 years or over. Similarly the annual incidence of 0.003% for those patients not consulting because of concern about pesticide exposure but for whom the GP thought their symptoms were likely to be related to pesticide exposure translates to an annual estimate of 6630 consultations i.e. about 128 per week for people aged 16 years or over.

### Home use of pesticides

547 (41%) interviewees reported using at least one pesticide in the home environment in the week before symptoms occurred (273 patients, 20% used 2 or more). The most frequently used pest control chemicals used at home in the week before symptoms occurred were slug and snail pellets and weed killer (table 2). For most of the chemicals in table 2 over half the patients reported that they had used it more than usual during that week. Almost a third of the pesticides were applied with an aerosol or spray, 25% as liquid and 21% as pellets or granules. 65.4% (358) of home pesticide users had used no personal protective equipment when applying the chemicals, although 284 patients (51.9%)) reported that their arms and legs were covered.

**Table 2:** Pest control chemical use at home

**Pest-control chemical**	**Number of Patients**	**Percentage of Pesticide Users**	**Reported more use than usual**	
			**Number**	**%**
**Weed killer**	131	23.9	74	56.5

**Kill root/nettles etc**	60	11.0	32	53.3

**Kill aphids/greenfly etc**	89	16.3	47	52.8

**Kill wasp/fly**	78	14.3	43	55.1

**Kill ant/cockroach etc**	80	14.6	57	71.3

**Fungicidal paint**	7	1.3	5	71.4

**Mould/mildew treatment**	48	8.8	30	62.5

**Tick/flea control**	98	17.9	44	44.9

**Head lice treatment**	17	3.1	11	64.7

**Insect repellent**	35	6.4	20	57.1

**Other animal repellent**	12	2.2	9	75.0

**Rat/mouse poison**	30	5.5	16	53.3

**Slug/snail pellets**	149	27.2	81	54.4

**Creosol/cuprinol**	57	10.4	42	73.7

**Dry rot treatment**	2	0.4	2	100.0

**Kill algae/lichen/moss**	15	2.7	7	46.7

**Intestinal worm treatment**	55	10.1	22	40.0

**Other**	22	4.0	15	68.2

In deciding on the quantity of pesticide to use 103 patients (44.6%) reported that they followed the label exactly, 103 patients (18.8%) used it as guidance, and 210 patients (38%) used their previous experience. The majority of pesticides stored at home were kept in the kitchen (15.7%) and/or in the garage or shed (63.3%).

61.5% of interviewees reported that they never disposed of pesticides and 25.5% disposed of them in the household rubbish bin. Relatively few reported that they used a chemical waste disposal site (2.2%) or other waste disposal site (7.1%).

The proportions of patients presenting with eye, skin, gastrointestinal or respiratory symptoms were almost identical for patients who used or did not use pesticides. A slightly higher proportion of the users had neurological and a slightly lower proportion of them had multiple symptoms (data not shown). However, almost twice as many of non users visited their GP because of flu symptoms compared with users.

The number of patients who reported some sort of change in use (more frequent use, change of brand, and/or larger quantity) of hazardous materials in the home other than pesticides was small. However, there was a slight tendency based on small numbers for an increased proportion of respiratory symptoms among patients who also used pesticides compared with those who did not use pesticides.

A follow-up questionnaire was sent to GPs after the study to investigate the criteria they used to categorize the symptoms of each presenting patient as possibly or likely to be related to pesticide exposure. It appeared that the decision by the GPs to use the category 'possibly related to pesticide exposure' was more often based on a discussion of both symptoms and activities (48% versus 41% of GPs), but less often based on pesticide alone (1% versus 19% of GPs) or included a discussion on specific pesticide exposure (10% versus 27% of GPs) compared to the decision to use the category 'likely'. Eligibility for invitation for an interview could thus have been biased by consideration of exposures, since those patients categorized by the GP as likely were automatically invited for an interview. For this reason logistic regression analysis was carried out excluding the likely group and those who consulted because of concern about pesticide exposure, thus comparing the risk of patients being categorised by their GP as having symptoms possibly related to pesticide exposure with those classified as unlikely to have symptoms related to pesticide exposure (1316 patients). Home use of pesticides and the change in use of several commonly used substances at home in the week before symptoms occurred showed statistically significant increased risk (Table 3 univariable results, based on 1316 patients categorised by the GP as having symptoms possibly related to pesticides).

**Table 3:** Univariable logistic regression models for the likelihood of being categorised by the GP as having symptoms possibly related to pesticide exposure

**Factors affecting pesticide related illness**		**Proportion of Participants (%)** **(n = 1316)**	**Odds Ratio** **(95% Confidence Interval)**
Occupational pesticide use versus no use		6	1.17 (0.62, 2.20)

Home pesticide use versus no use		41	1.83 (1.49, 2.26)

Age (one year increase)		-	0.99 (0.985, 0.997)

Male versus female		42	1.0 (0.81, 1.24)

Proximity of farmland	100 m-1 km vs < 100 m	29	0.65 (0.45, 0.93)
	> 1 km vs < 100 m	40	1.79 (0.55, 5.83)
	Don't know vs < 100 m	2	0.59 (0.26, 1.36)

Proximity of chemical plant	100 m-1 km vs < 100 m	2	0.91 (0.19, 4.32)
	> 1 km vs < 100 m	62	1.23 (0.25, 6.05)
	Don't know vs < 100 m	35	0.69 (0.19, 2.49)

Proximity of landfill site	100 m-1 km vs < 100 m	3	2.92 (0.81, 10.49)
	> 1 km vs < 100 m	80	1.84 (0.39, 8.68)
	Don't know vs < 100 m	16	1.38 (0.30, 6.35)

Proximity of heavy traffic	100 m-1 km vs < 100 m	37	0.93 (0.66, 1.31)
	> 1 km vs < 100 m	39	0.80 (0.46, 1.37)
	Don't know vs < 100 m	1	0.53 (0.12, 2.23)

Proximity of railway	100 m-1 km vs < 100 m	30	0.96 (0.65, 1.43)
	> 1 km vs < 100 m	58	1.41 (0.70, 2.84)
	Don't know vs < 100 m	4	0.84 (0.27, 2.63)

Area of living	Surburban versus urban	37	0.29 (0.08, 1.02)
	Rural versus urban	39	0.29 (0.07, 1.1)

Change in use of Laundry detergent		9	1.60 (0.93, 2.76)

Change in use of Disinfectant/bleach		8	1.26 (0.81, 1.95)

Change in use of Cleaning agent		8	1.43 (0.88, 2.34)

Change in use of White spirit		9	1.60 (1.06, 2.41)

Change in use of Polish/varnish		6	1.83 (0.92, 3.66)

Change in use of Air freshener		10	1.25 (0.89, 1.77)

Change in use of Paint		11	1.74 (1.19, 2.54)

Change in use of Toiletries		7	1.85 (1.22, 2.80)

Change in use of Stain remover		4	1.36 (0.63, 2.91)

Change in use of Furniture renovator		4	1.86 (1.19, 2.93)

Change in use of Oil/grease		3	1.12 (0.49, 2.57)

Change in use of Insulation material		5	1.06 (0.64, 1.74)

In multivariable models that included occupational and home use of pesticides, age and gender, and change in use of each of the 12 commonly used substances in Table 3 in turn, none of the 12 substances altered the odds ratios for occupational or home use of pesticides substantially (ORs for occupational pesticide use ranged from 0.97 to 1.01 and ORs for home use of pesticides ranged from 1.83 to 1.88). These variables do not appear therefore to be confounding the effect of occupational and home pesticide use. The risk associated with changed use of paint, toiletries and furniture renovator remained significantly raised in these analyses, although based on small numbers.

The results of a multivariable model including occupational and home use of pesticides, age as a continuous variable and gender are given in Table 4 and show a statistically significant increased risk for being categorised as having possible pesticide related symptoms if the pesticide was used at home (OR = 1.88, 95% CI 1.51 – 2.35) (Table 4).

**Table 4:** Multivariable logistic regression model for the likelihood of being categorised by the GP as having symptoms possibly related to pesticide exposure

**Factors affecting pesticide related illness**	**Multivariable model** **OR (95% CI)**
Occupational pesticide use versus no use	0.99 (0.53, 1.88)

Home pesticide use versus no use	1.88 (1.51, 2.35)

Age (1 year increase)	0.99 (0.99, 1.00)

Male versus female	1.02 (0.81, 1.28)

## Discussion

The results from this study suggest that the incidence and prevalence of ill health presenting to and diagnosed in Primary Care as related to pesticides in GB are small but that this could result in considerable numbers of consultations. There are no directly comparable studies in the primary care sector in GB. In 2005/2006 there were 169 hospital episodes of accidental poisoning by exposure to pesticides in the UK, 93% of which were emergency admissions and 70% occurred to children under the age of 15 years [5].

Other countries also monitor acute pesticide poisoning and there has been some attempt by the World Health Organization to provide a standardised case definition and classification scheme with regard to exposure, health effects and causality [9]. Underreporting of acute events can occur in monitoring systems, as illustrated in a cross-sectional survey Nicaragua [10]. Pesticide-related illness as an important cause of acute morbidity among migrant workers has also been found in multi-state standardised occupational surveillance programs such as those led by the US National Institute for Occupational Safety and Health which include primary care physicians [11].

In designing our study we found few UK based statistics on which to base sample size calculations but anticipated that the incidence of possible pesticide-related illness could potentially be as low as 1% (as indeed was the case, 1.6%) An early study on deaths from pesticide poisoning in England and Wales indicated that such deaths represented 1.1% of all deaths from poisoning over 1945–1989 [12] and at least 73% of these pesticide fatalities were due to suicide. We originally aimed to recruit 70 practices from the GPRF giving access to approximately 420,000 patients, about 0.75% of the UK population but, although the GPRF network consists of practices interested in participating in research, it proved difficult to recruit them. We did, however, manage to recruit 43 Practices with over 160 GPs and nurse practitioners participating in the study.

These practices were well spread geographically throughout GB between urban, suburban and rural areas and between different areas of deprivation, with the average list size per GP in the practices being 2210, varying from under 1000 in a rural area to over 2500 in 2 city practices. The average list size of GPs during our study period in England and Wales was 1666 [13]. The average number of surgeries held per week per practice, used for the pesticide project in the participating practices, was 2.4 over the study period. In the UK generally the average number of surgery sessions held weekly by GPs is about 8. The study thus included about 30% of the consulting workload of each participating GP. Overall, of all those patients screened, 15.5% of those not asymptomatic (6930 of 44829 patients with symptoms) had a respiratory problem (including flu-like symptoms). The same proportion of patients was estimated to consult their GP for respiratory condition problems in the UK in 2002 [13]. The corresponding figures from our study and for the UK respectively are: skin symptoms 12.2%, 10.9%; eye problems 2.2%, 4.5%; gastrointestinal (our study)/digestive system (UK figures) 8.7%, 7.2%; neurological (our study)/nervous system (UK figures) 1.8%, 3.4%. Although in our study all participants were over 18 years old and the UK estimates include all ages the similarity of these figures suggests that our study closely mirrors the general pattern of symptoms within the UK consultations.

Over 40% of those interviewed in our study had use a household pesticide in the week before symptoms developed. Other studies have tended to ask about past use of household pesticides over a longer period. For example, a case-control study of childhood haematopoietic malignancies in France found that about 50% of mothers of cases had used a household pesticide during pregnancy; use varied by type of residence and area of residence e.g. for fathers use during pregnancy was 72%, 61% and 44% for rural, mixed and urban areas respectively and 86%, 67% and 26% for living in a farm, house and flat respectively [14]. A similar study of childhood leukaemia found that more than half of households had used insecticides or indoor pesticides during the first year after the birth of the child [15]. A UK survey of a sample of parents from the Avon Longitudinal Study of Parents and Children found that 93% had used at least one pesticide product in the last year with a range of frequencies [16]. For example, 33% of parents used pesticides to treat pets with a median use of 4 times per year; other use included insecticides (42% of parents, 1–90 applications per year, median 2.5), slug pellets (44% of parents, 1–60 applications per year, median 4), weed killer (27% of parents, 1–18 applications per year, median 2). A UK observational study found that few participants read the label, that they often found it hard to understand and that compliance with instruction was low [17].

Our study is limited in some aspects. It was felt that it would be too impractical and costly to try and interview in depth a random sample of patients consulting their GPs throughout a year at a large number of practices. We also wanted to gain some knowledge of GP diagnosing of pesticide-related illness. The screening checklist was thus designed to include this and to screen out patients who were asymptomatic, consulting for on-going/chronic health problems or whose symptoms, in the opinion of the GP, were definitely not related to pesticide exposure. This reduced the proportion of patients eligible for an interview to 8%. Some of those not invited for interview could have had a pesticide-related problem not attributed by either the patient or GP to pesticides, leading to potential underestimation of incidence and prevalence.

At the completion of the study a follow-up questionnaire was sent to GPs (86 GPs from 32 of the 43 practices responded) to investigate how they decided on their classification of the likelihood of pesticide-related illness. 71 GP (83%) of the respondents classified patients to have possibly pesticide related illness on the basis of symptoms and/or activities before the symptoms occurred. Only one GP reported using only a mention of pesticides to categorise a patient as 'possible case'. These results confirm that very few GP specifically discussed pesticides with the patient when categorising them as 'possible related to pesticide exposure'.

A high proportion (44%) of those invited for interview refused to participate and this did not improve after we had received ethical approval to carry out telephone interviews. However, overall, 50% of those who did not refuse were interviewed. The interview questionnaire was fairly lengthy. It was felt important to consider total exposure to pesticides from all sources. The interview thus attempted to capture these data. The information on actual chemicals and active ingredients of pesticides is, however, limited as it was thought that patients would either not know this or be unable to recall it accurately. The study was thus limited in its ability to define a definitive pesticide-related case of ill- health. However, other systems such as the SENSOR system in the US and the Pesticides Incident Reporting Scheme in the UK also use some element of self-reporting and expert judgement, particularly in defining a possible case [18,19]. In addition we did not, in this study, follow up patients categorised as having likely or possible pesticide-related illness with regard to treatments or the results of this. The establishment of a definite causal relationship from these systems, as in our study, would thus require careful consideration.

A clear outcome from the study is that it would not be feasible to implement the methods used in this study for a wider surveillance system in GB. Screening of patients at nearly 60,000 consultations yielded relatively few likely or probable cases of pesticide-related symptoms. The project also required constant monitoring, and motivation and encouragement of the practices. This would be infeasible as part of a routine monitoring system.

Throughout the world primary care surveillance networks have been developed that monitor voluntarily one or more specific illness problems on a regular or continuing basis [20,21]. The main objective of these is on disease surveillance, many focussing on infectious diseases nationally or as part of international networks such as GeoSentinel and TropNetEurope [22,23]. Primary Care physicians may also detect sentinel cases of occupationally or environmentally caused diseases. For example, a cluster of Guillain-Barre syndrome cases was observed in relation to aerial organophosphate insecticides [24].

In the UK, it has been shown that none of the existing GP morbidity recording schemes routinely recorded occupation although it would be feasible to add procedures to obtain this information [25], as it would for environmental factors. The importance of incorporating environmental health into primary care education and practice has been recognised in other countries. In the US, the National Strategies for Health Care Providers: Pesticides Initiative and the national Environmental Public Health Tracking program have been launched [26,27]. The former aims to raise awareness among GPs and nurses of potential exposures to pesticides. Cities like New York are investigating how to develop their capacities to track and link environmental public health indicators such as pesticide sales and applications, housing and building information and medical data. However, data collected for surveillance need to be relevant and action-driven [28]. In many studies worldwide there has been recognition of the under-reporting of pesticide related poisoning and the need to improve surveillance [29]. Although the prevalence and incidence results from our study are small these potentially give fairly large number of patients presenting to GPs each week with possible pesticide related illnesses, albeit mild ones. Our study has thus shown that there is a need to extend surveillance to include Primary Care.

## Conclusion

The use of pesticides was widespread among interviewees in the home environment and there was unsatisfactory use of product labels and precautionary measures; storage and disposal of pesticides was also poor. Although the estimated annual prevalence and incidence of pesticide related illness reported to and diagnosed in Primary Care was small, this implies large numbers of consultations potentially concerning pesticide exposure. The study methods used here are not feasible for routine pesticide illness monitoring system. However, there is a need to incorporate environmental health into Primary Care education and practice.

## Competing interests

The authors declare that they have no competing interests.

## Authors' contributions

LR was one of the initiators and had overall management of the study and participated in its design and coordination. VM participated in the design and conduct of the study and performed the statistical analysis. Both authors contributed to the drafting of the manuscript and approved the final version.

## Pre-publication history

The pre-publication history for this paper can be accessed here:



## Supplementary Material

Additional file 1 Checklist and questionnaire. Click here for file
