# Supplementary material for: Pesticide-related illness reported to and diagnosed in Primary Care: implications for surveillance of environmental causes of ill-health
Source: BMC Public Health. 2009 Jul 6;9:219. doi: 10.1186/1471-2458-9-219 (PMC2718882; doi:10.1186/1471-2458-9-219)
Supplement: Additional file 1 — Checklist and questionnaire. [file 1471-2458-9-219-S1.doc]

Pesticide-related illness reported to and diagnosed in Primary Care: implications for surveillance of environmental causes of ill-health

Lesley Ruston, Vera Mann

1. GP Checklist

2. Computerised Interview Questionnaire

# 1. GP CHECKLIST

# General practitioner based scheme for monitoring pesticide related illness

###### Name of Patient________________________________ DoB ______________________

Today’s date_____________________ AM or PM GP Initials______________________

*(Please Circle)*

##### No Yes

1. **Is this patient consulting because of exposure only?**
2. Is this patient consulting because of exposure

and related symptoms?

3 Is this patient symptomatic (for any reason)?

4 Has this patient previously consulted for this problem

within the last 3 days?

1. Does this patient have serious and acute symptom/s?

(e.g. blurring of vision, vertigo, respiratory compromise etc)

1. Does this patient have one or more of the following

symptoms? (please tick all that apply):

- **Flu type symptoms**
- **Respiratory**
- **Gastrointestinal**
- **Skin**
- **Eye**
- **Acute neurological**
- **None of the above**

**No Yes Don’t know n/a**

1. Do you think the presenting symptoms are unusual for

**this particular patient?**

**Likely Possible Unlikely Definitely not n/a**

1. In your opinion, how likely is it that the patient’s

**symptoms are related to pesticide exposure?**

**If eligible, is there any reason why this patient should not be invited to join the study? YES**

# 2. SCREENS FROM COMPUTERISED INTERVIEW QUESTIONNAIRE


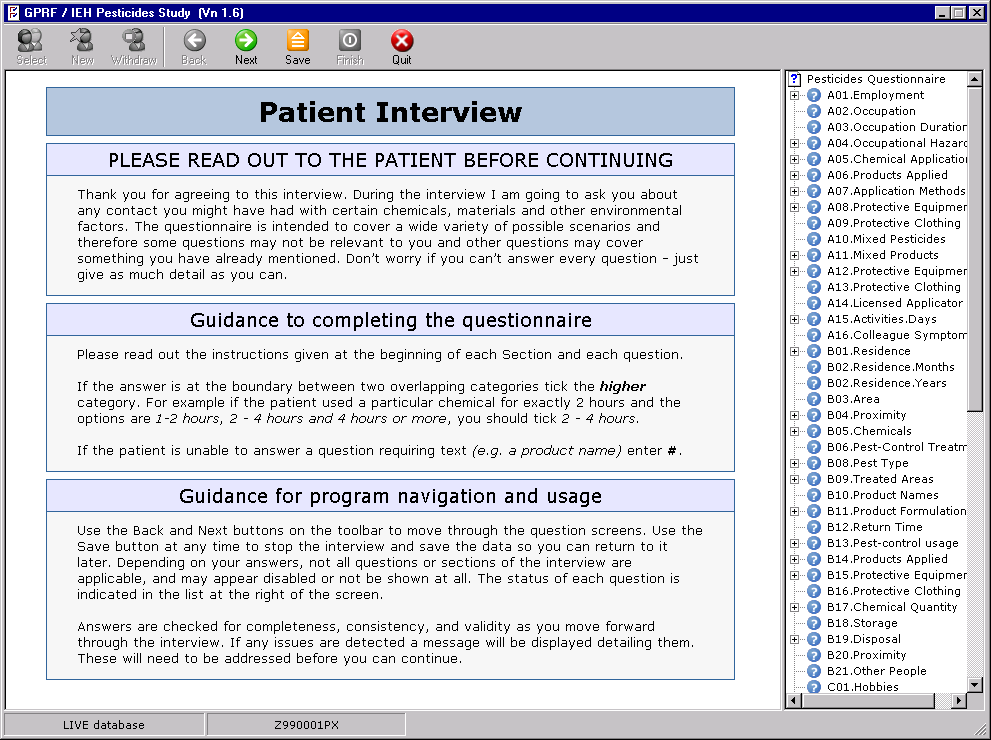


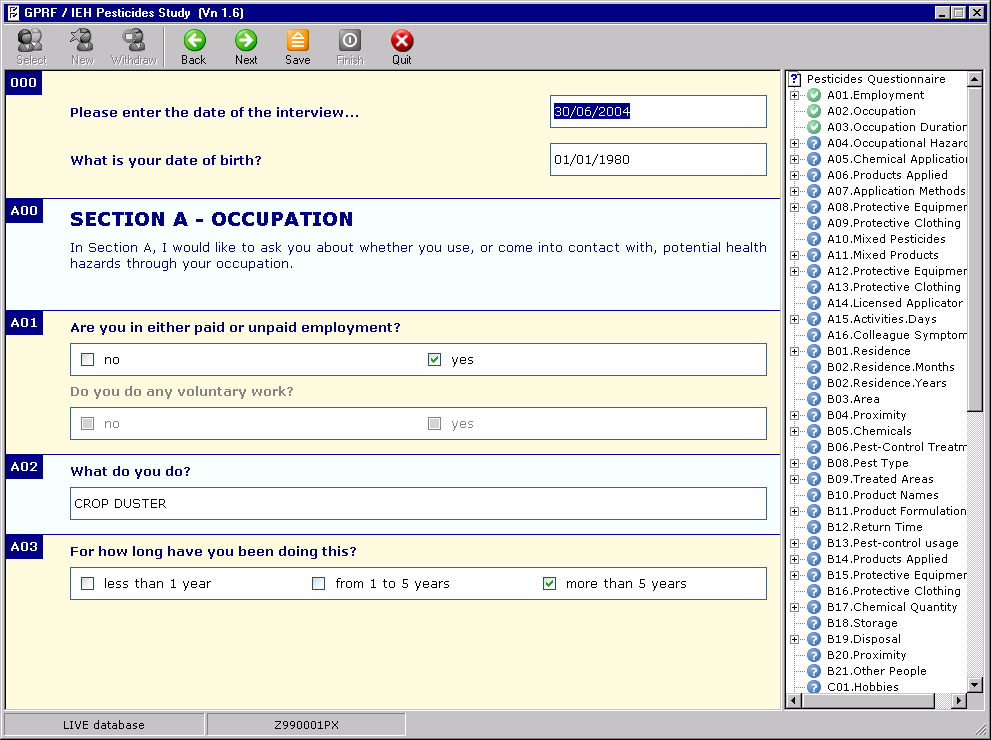


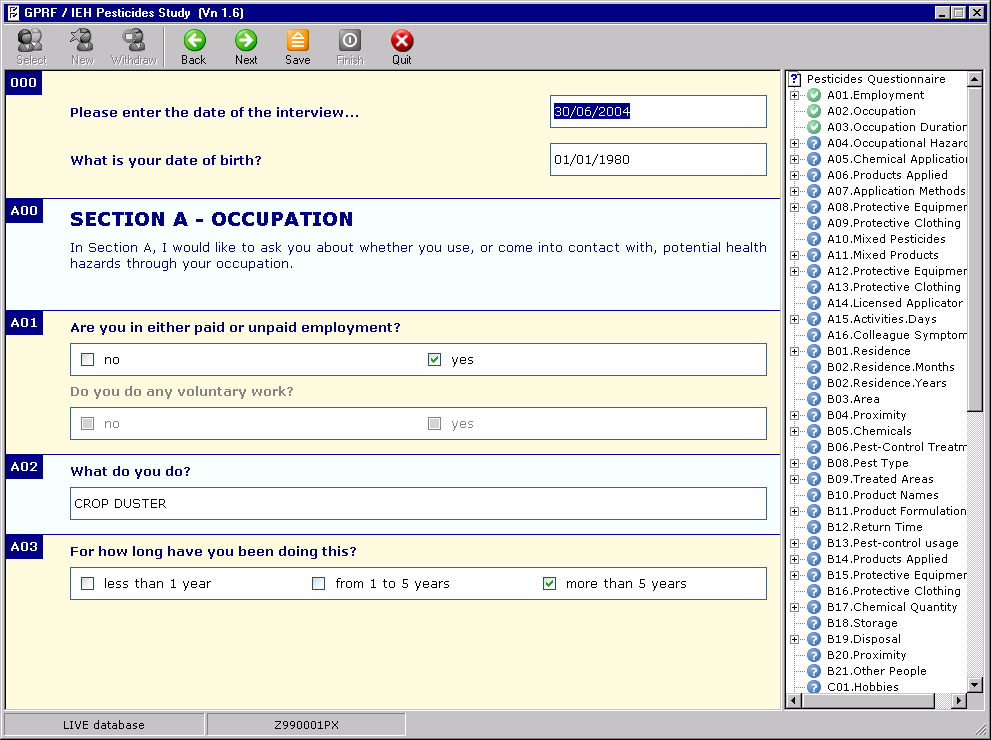


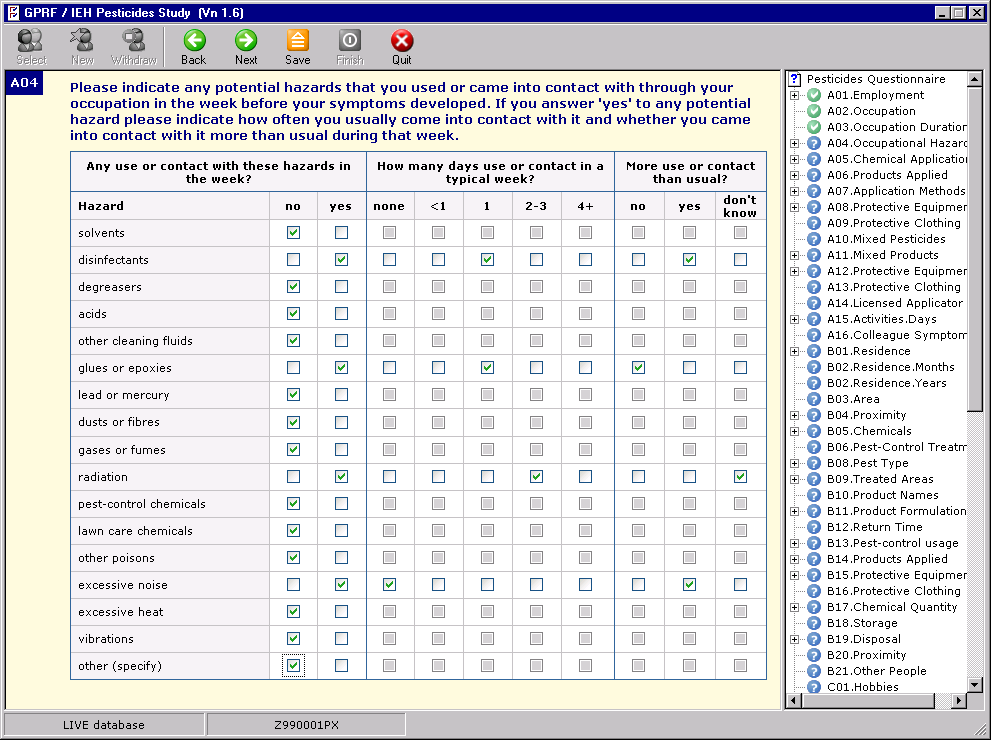


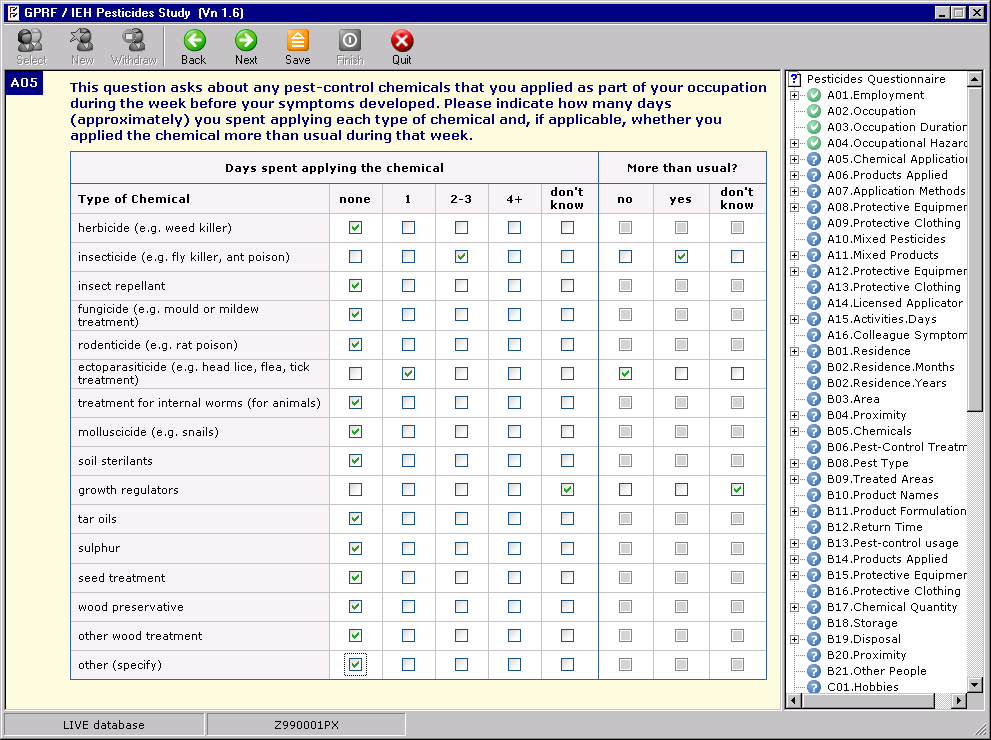


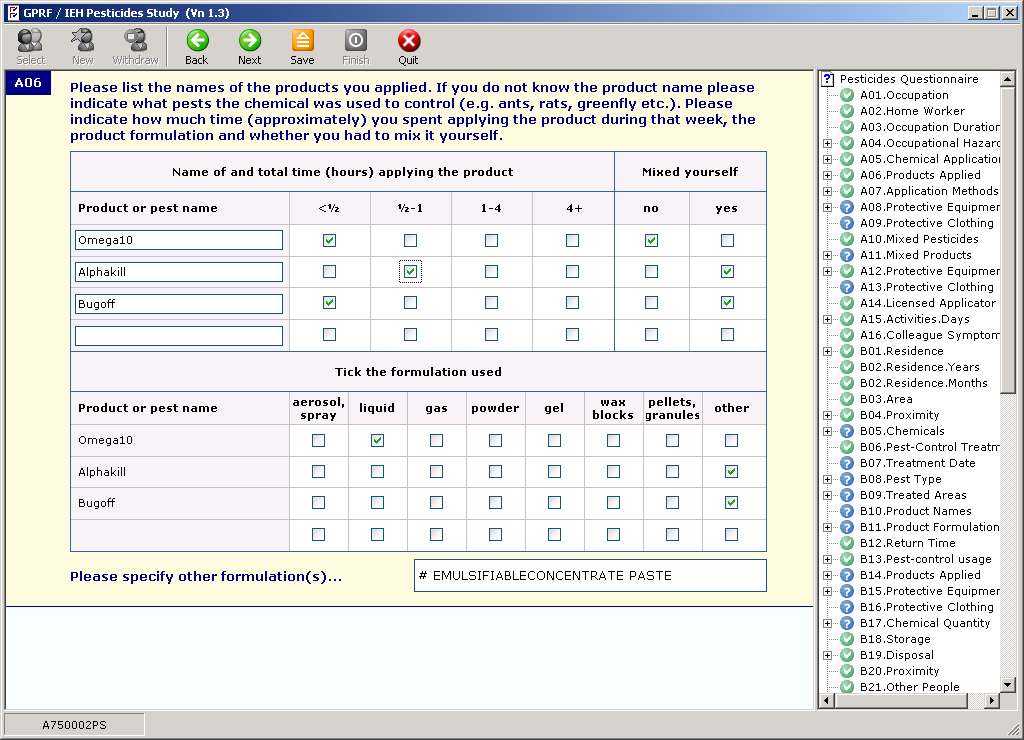


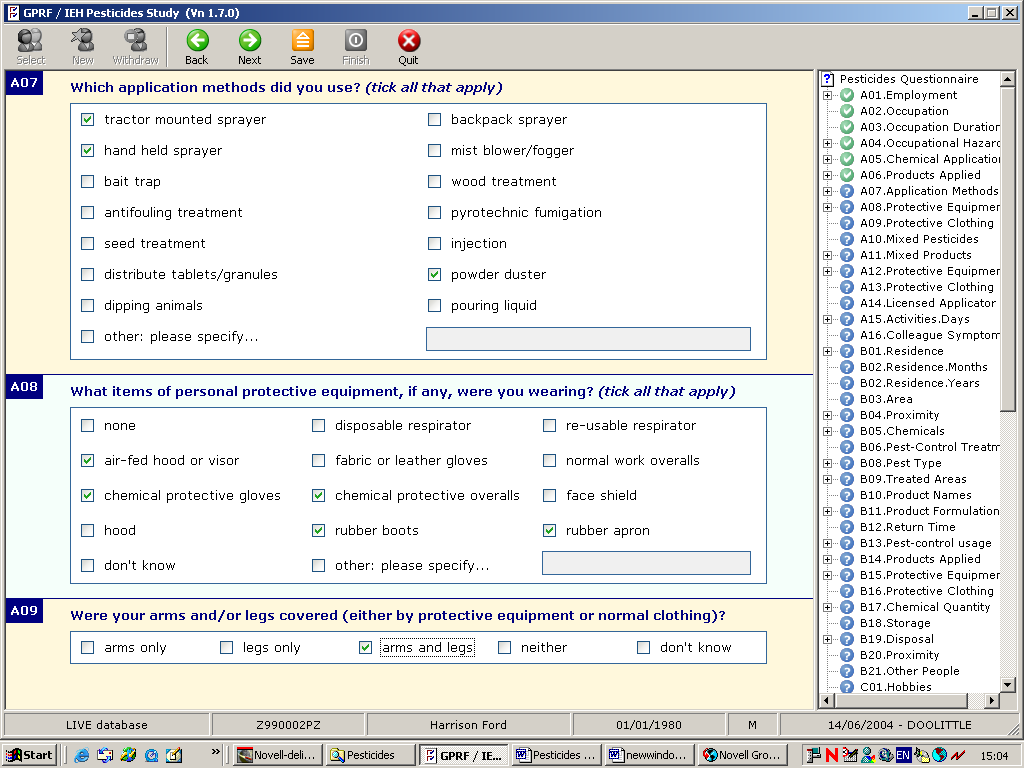


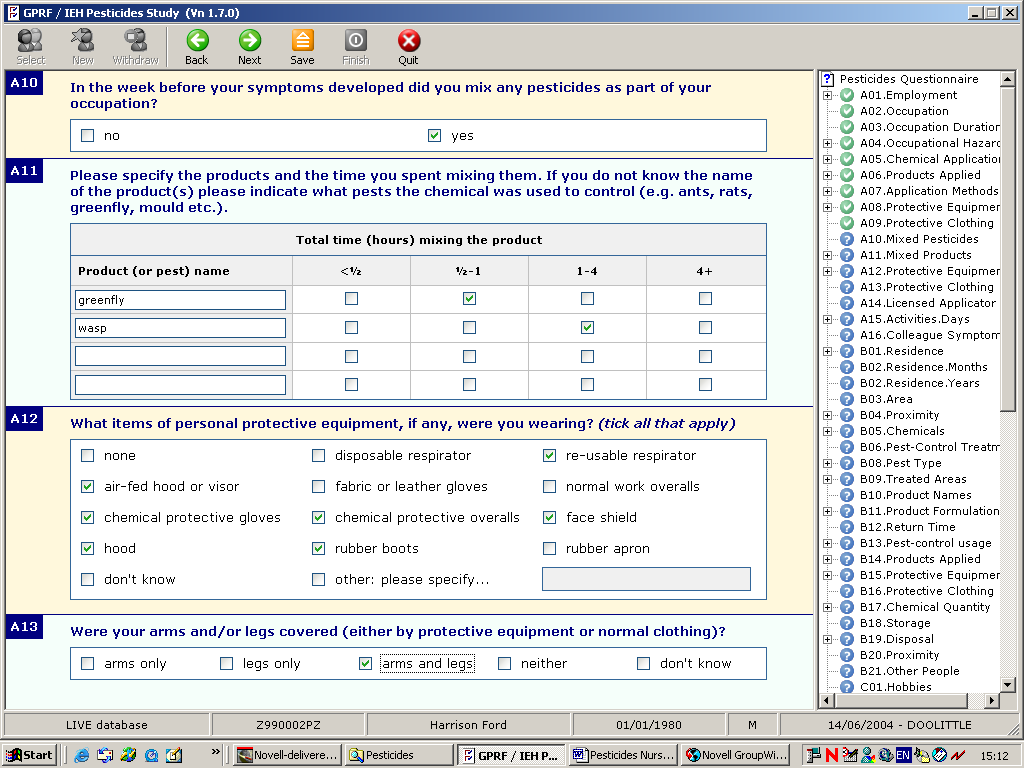


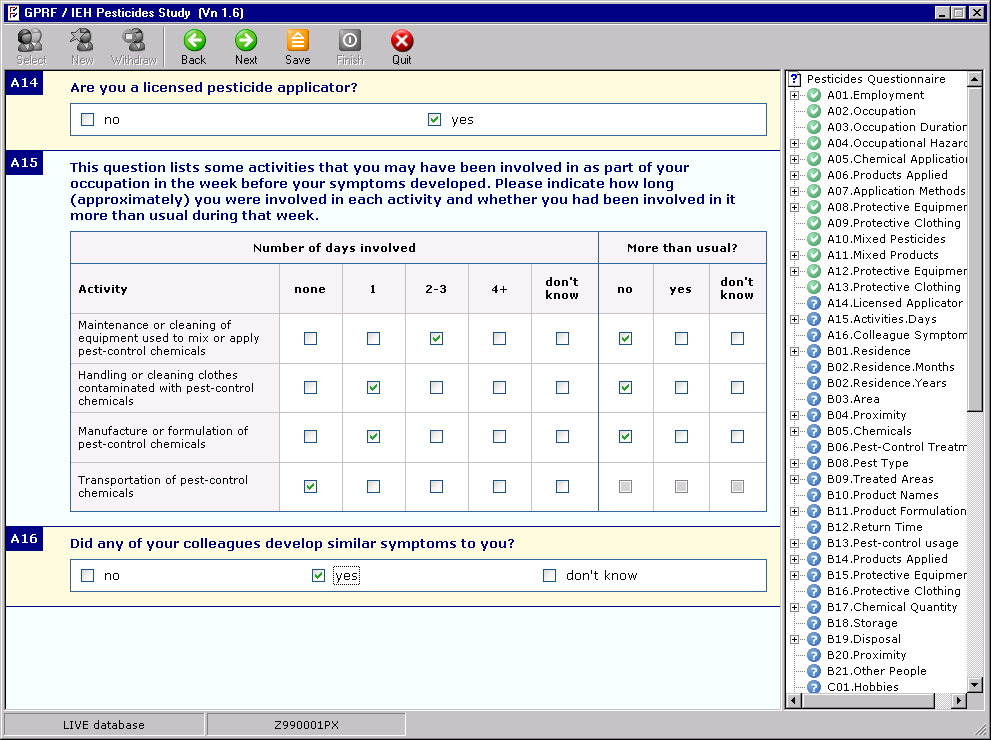


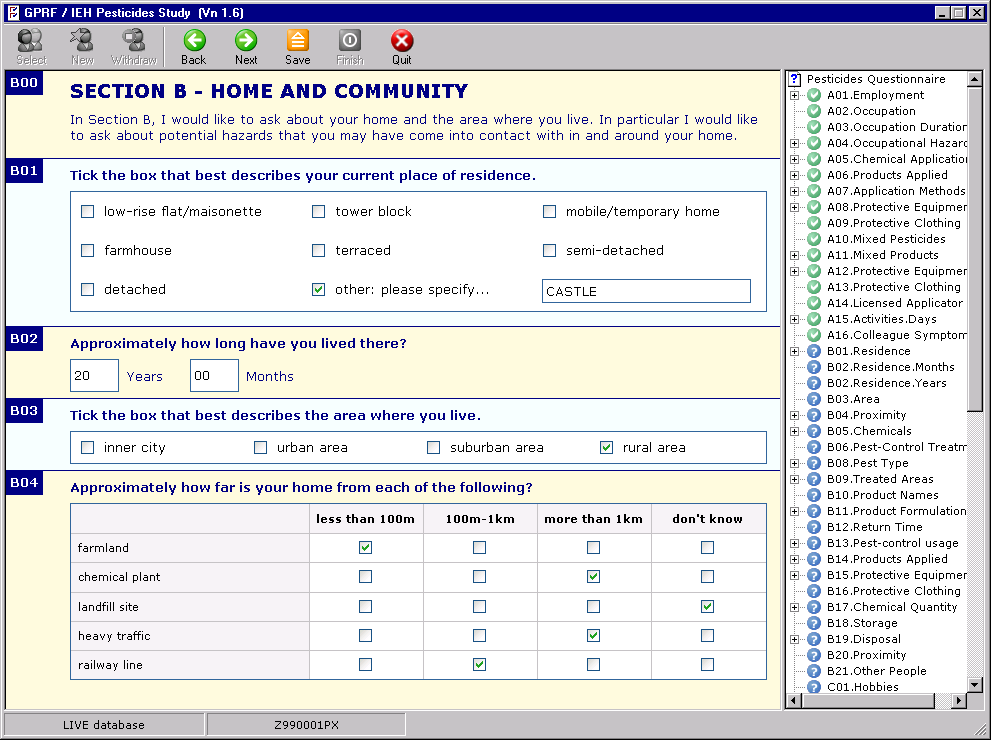

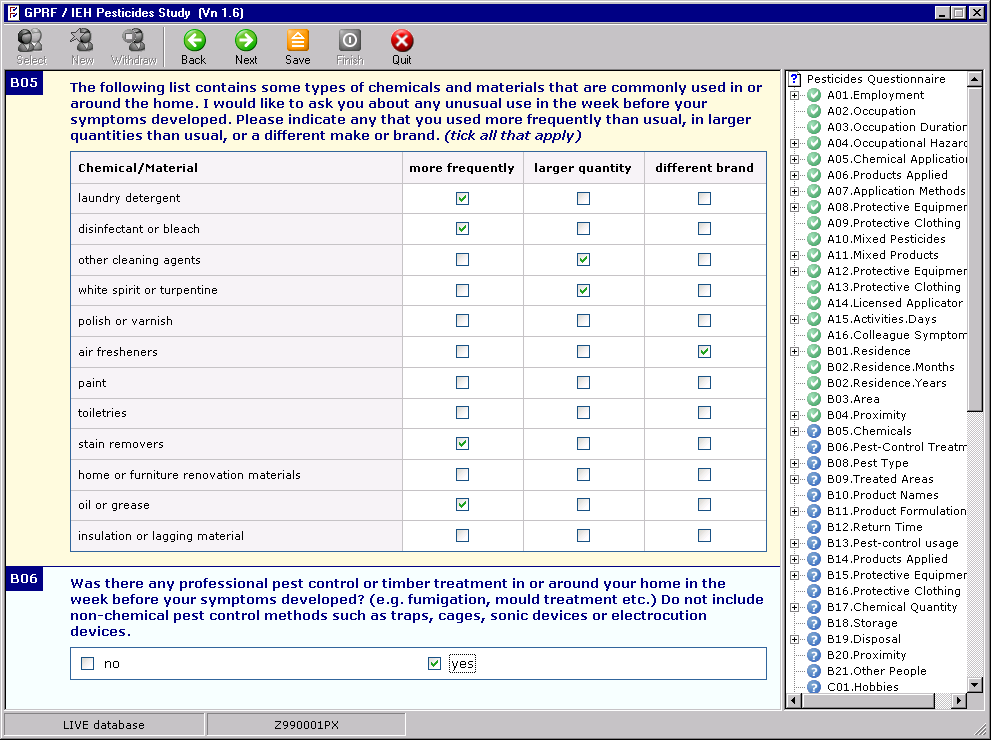


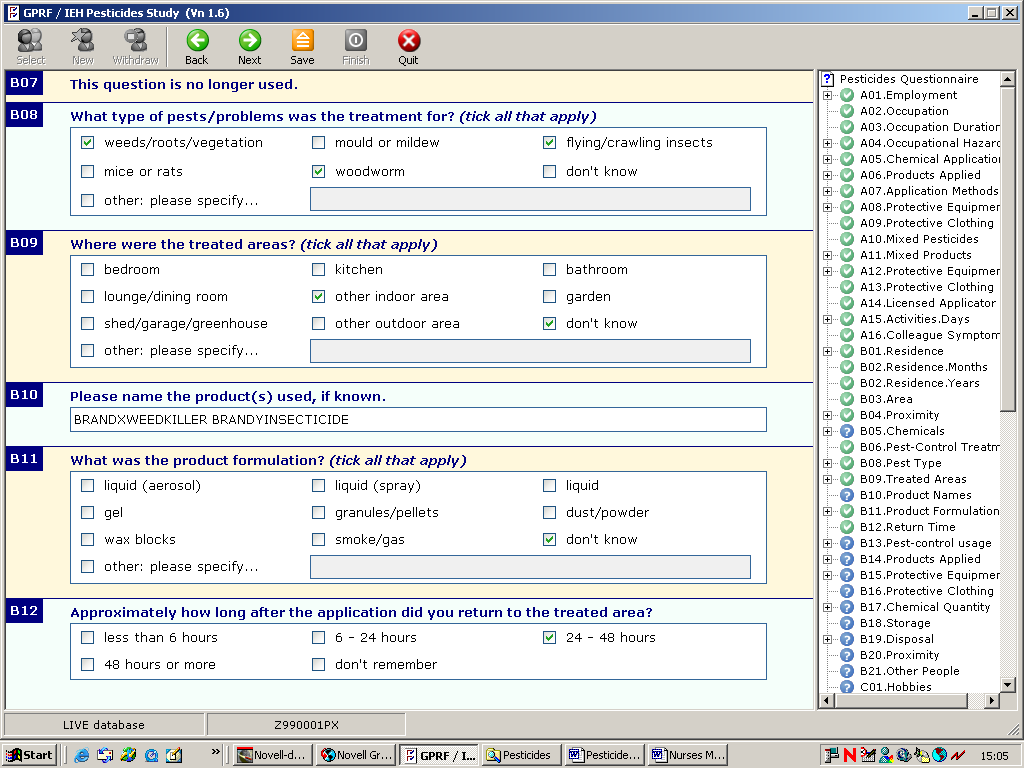


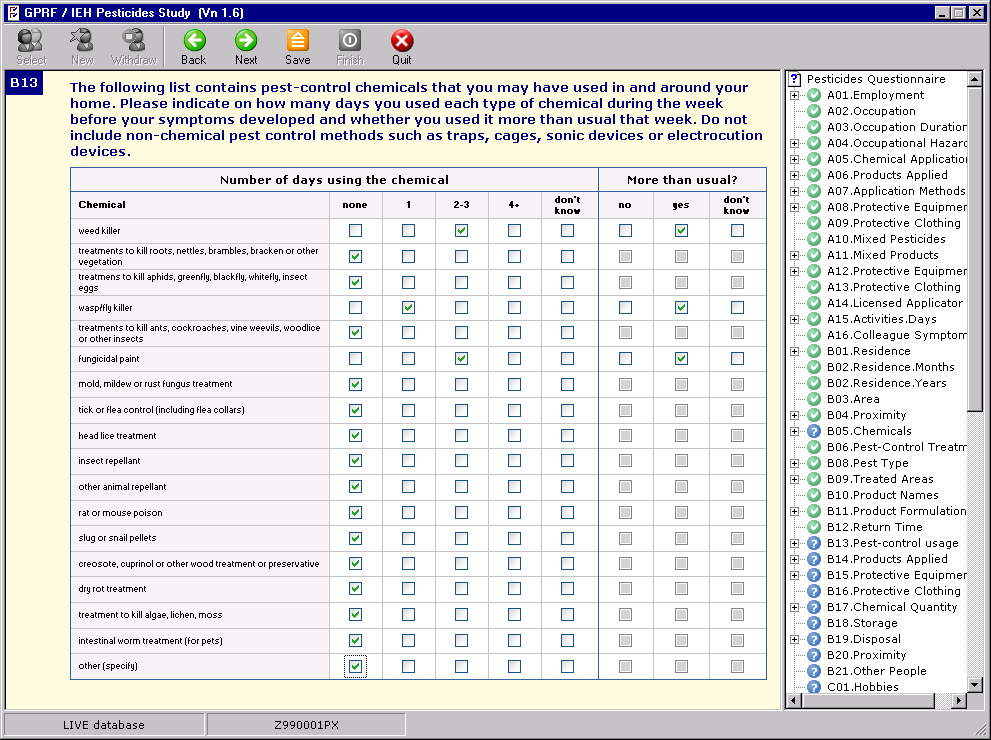


**
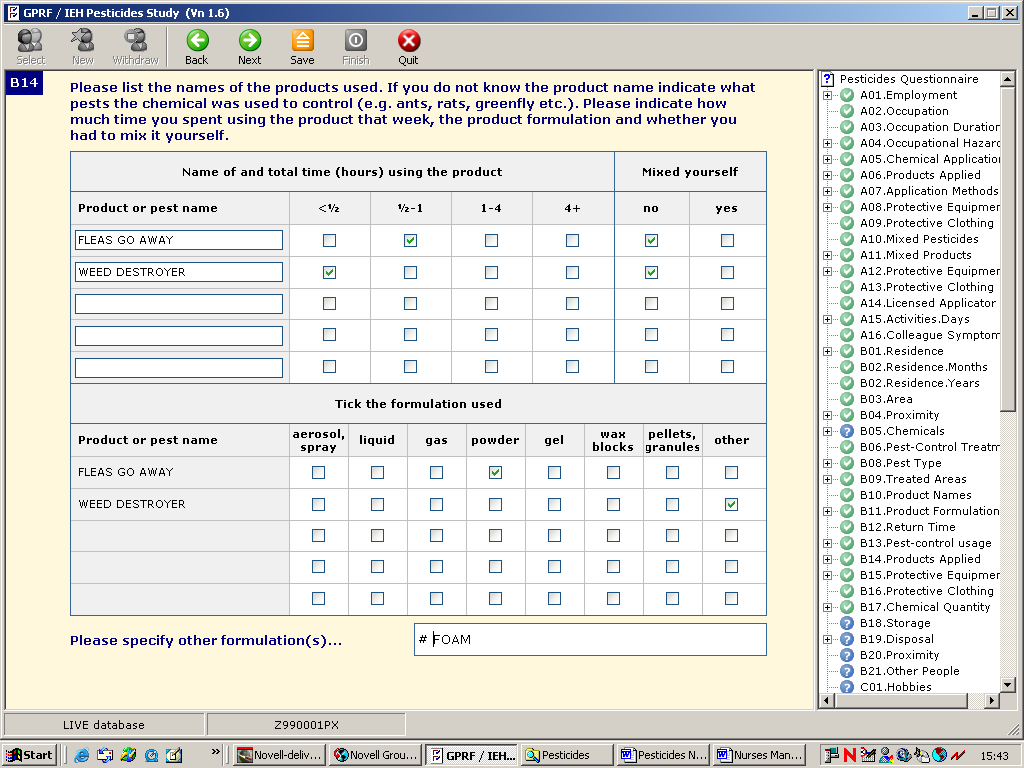
**


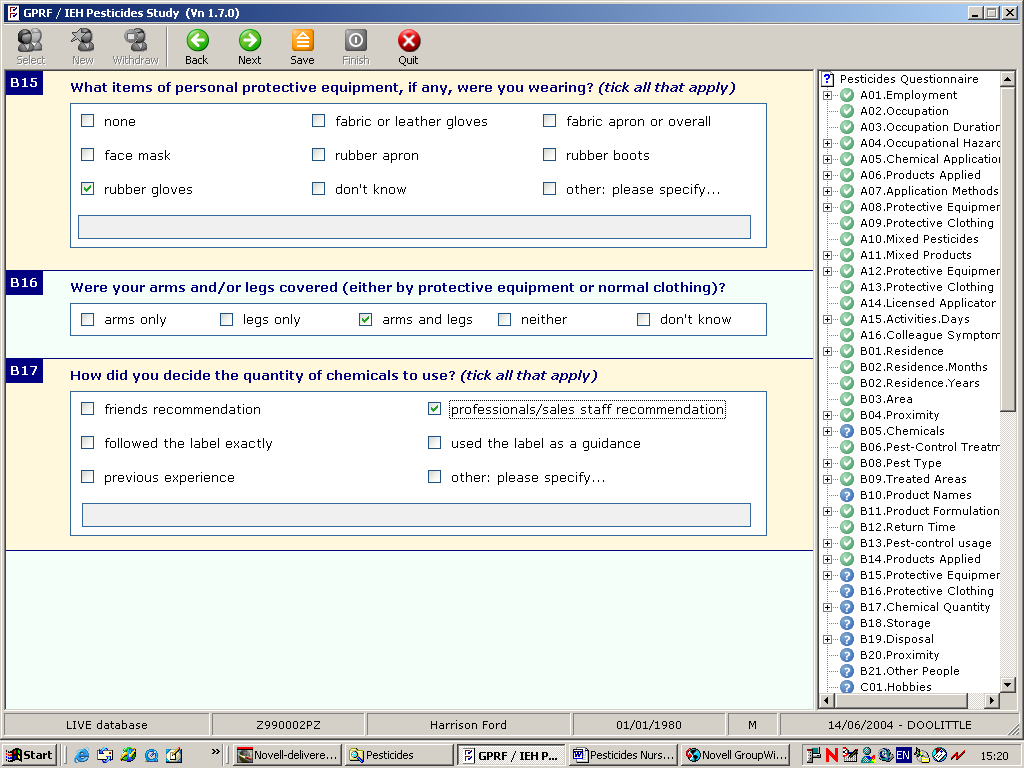


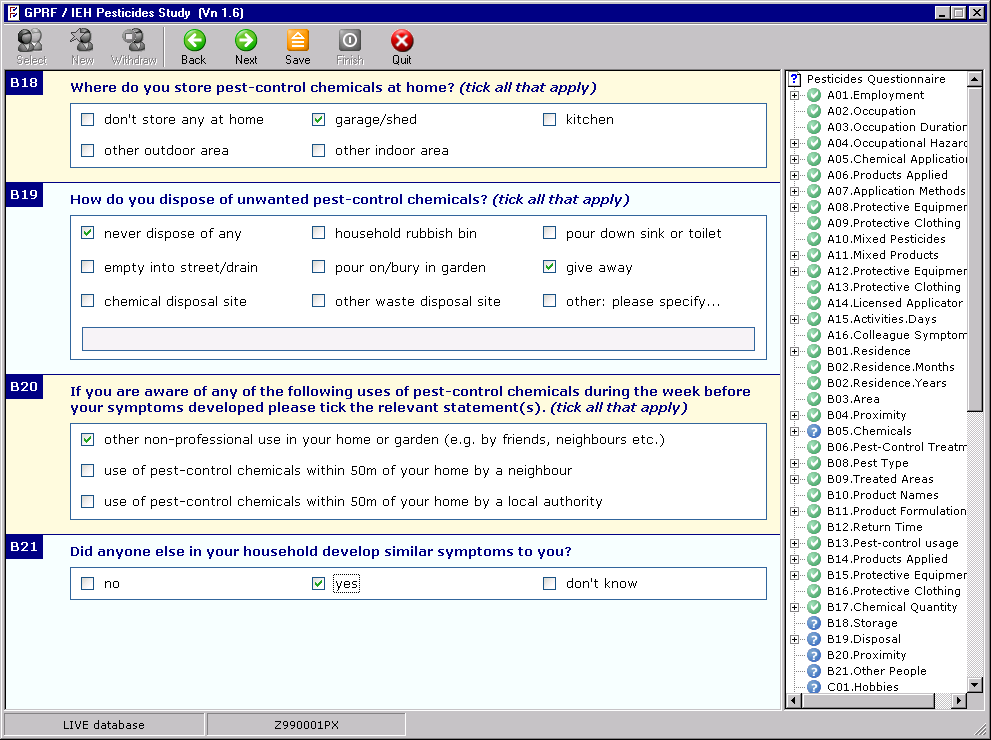


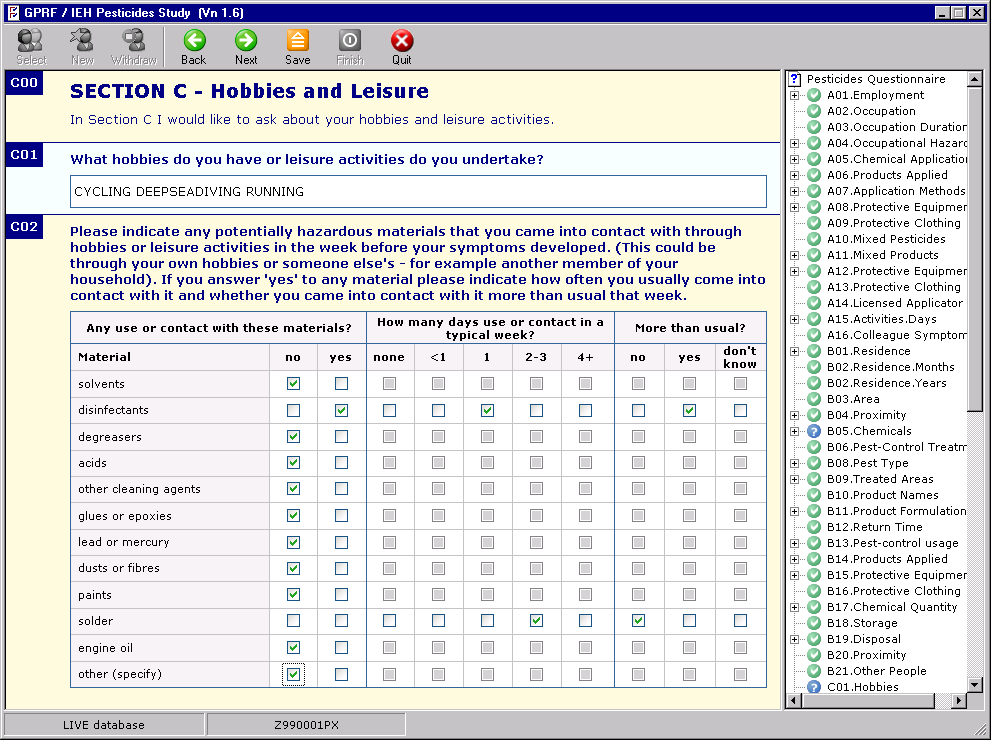


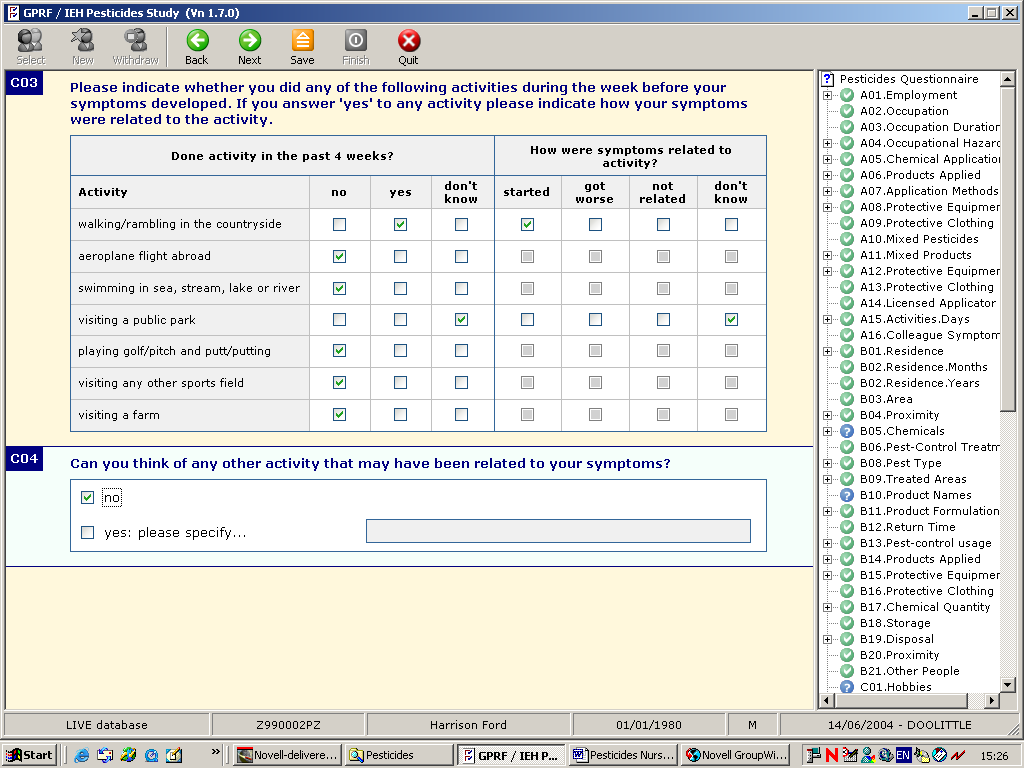


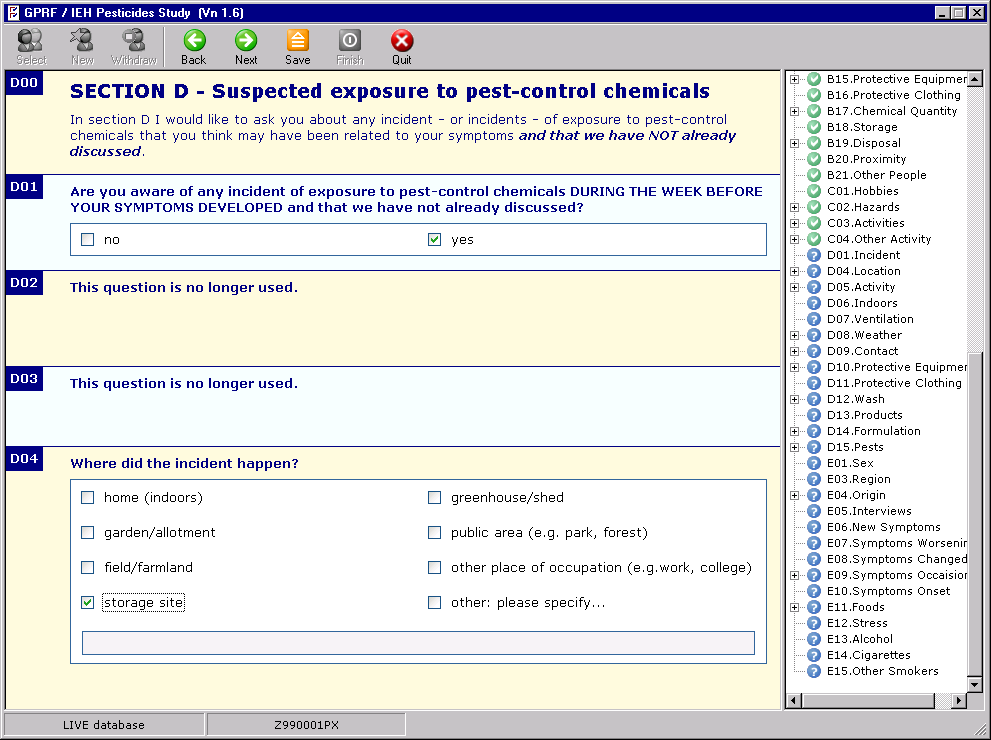


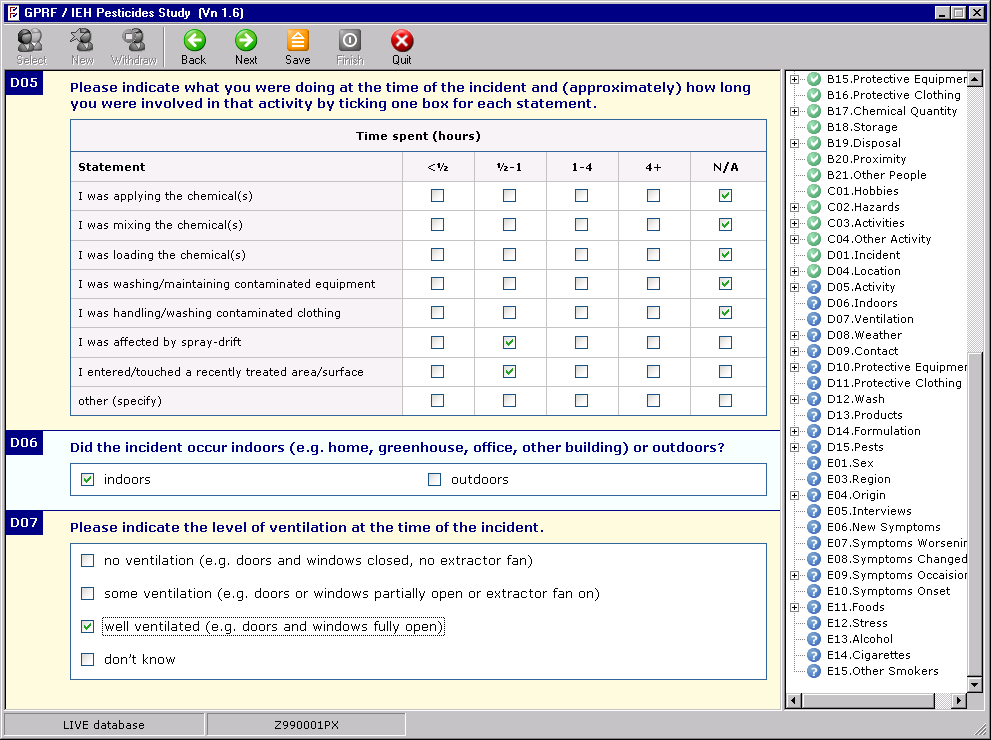


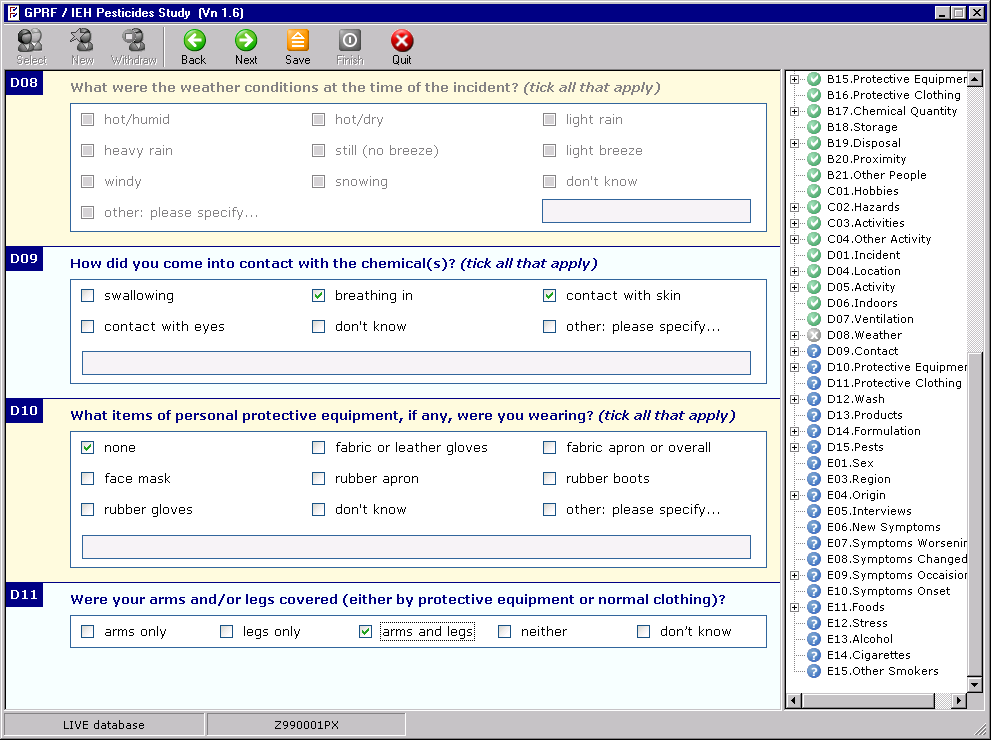


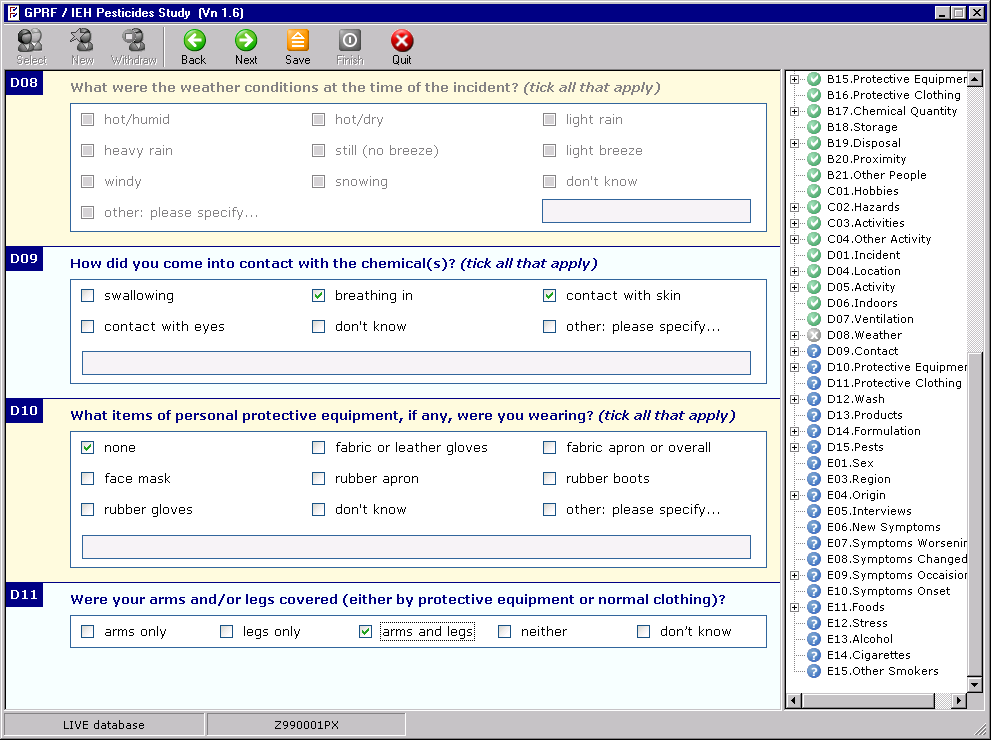


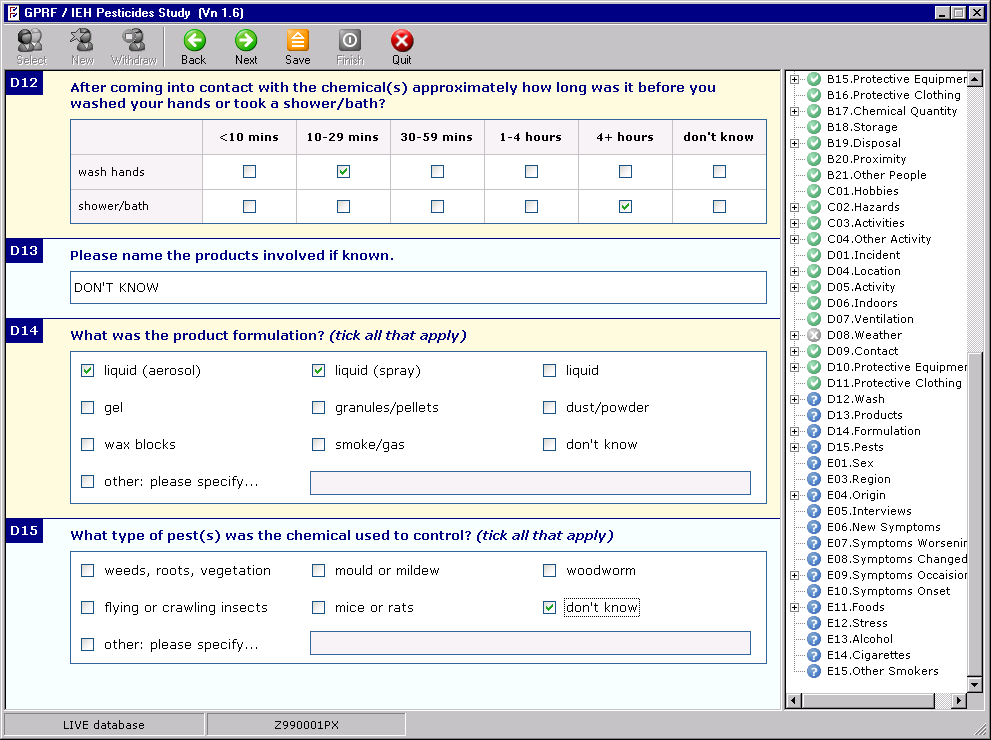


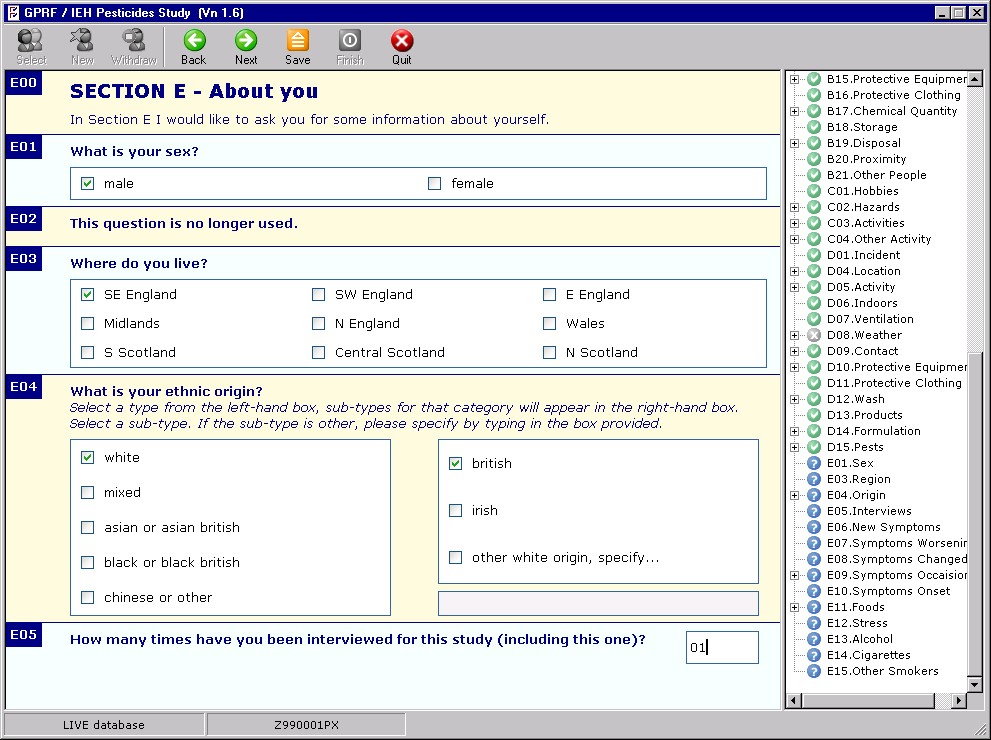


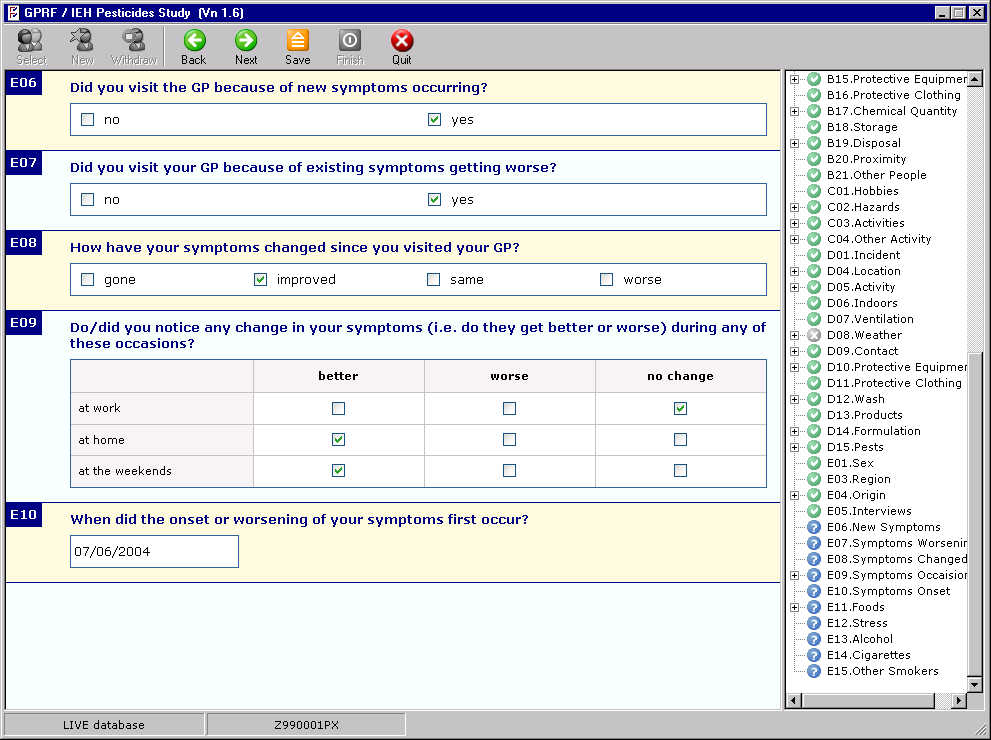


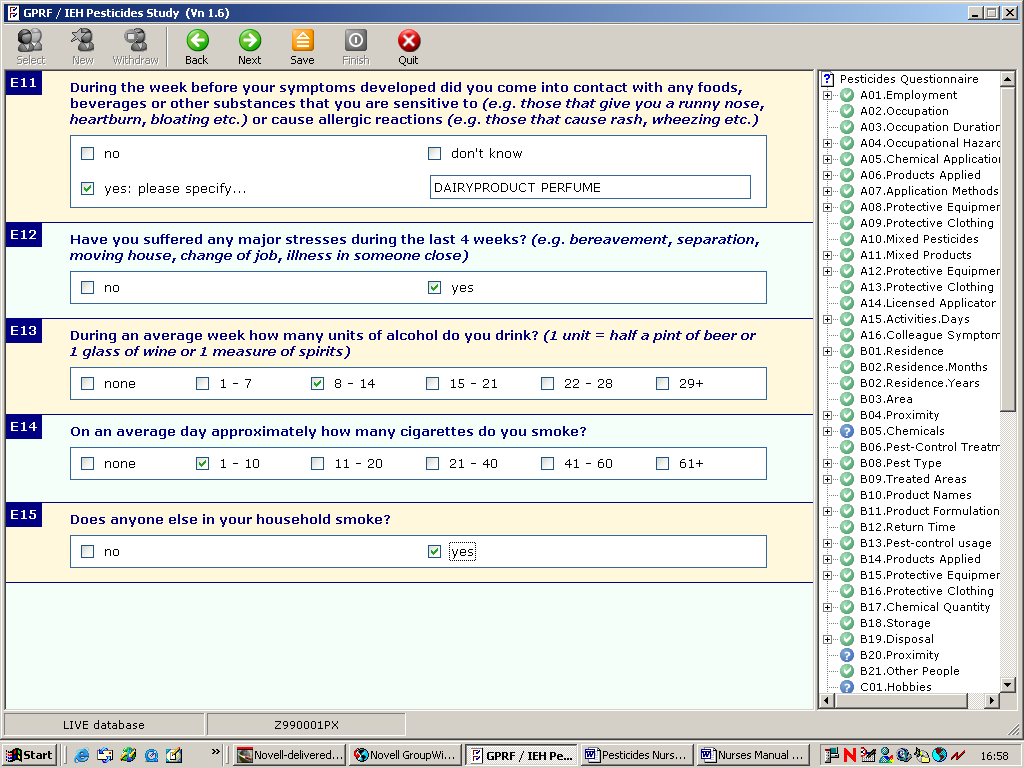


**
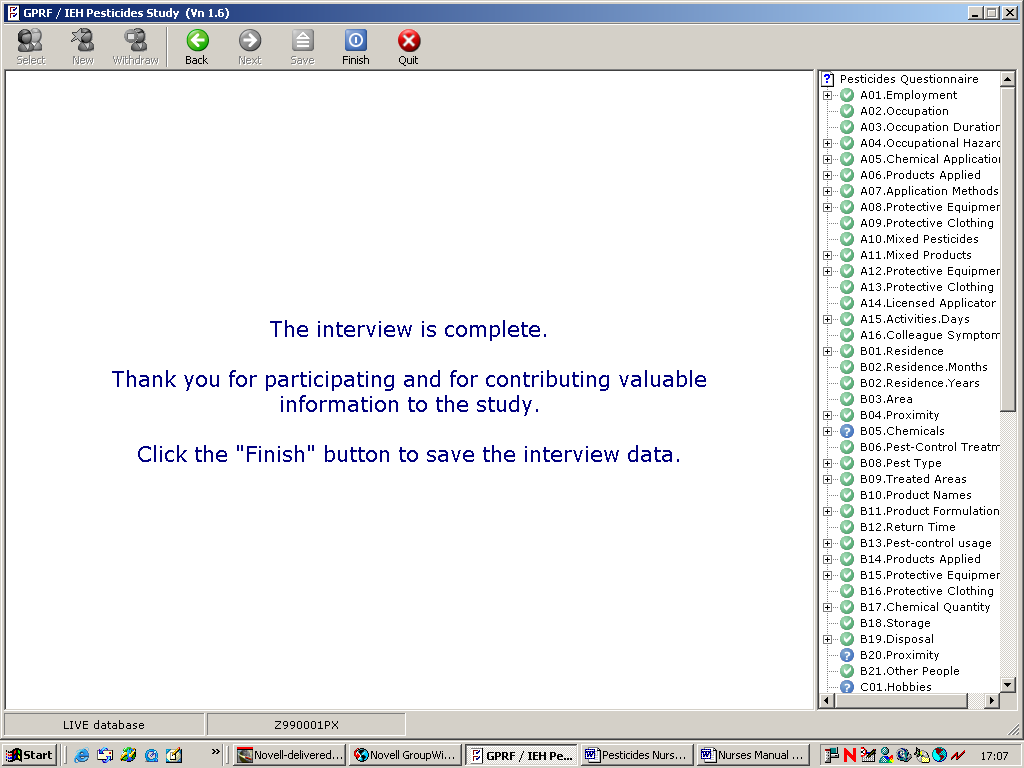
**
